# A Public Address on the Advancement of Medical Science

**Published:** 1858-12

**Authors:** O. Q. Herrick


THE CHICAGO

### MEDICAL JOURNAL.

VOL. I. DECEMBER, 1858. No. 12.

### ORIGINAL COMMUNICATIONS.

ARTICLE I.

### A PUBLIC ADDRESS ON THE ADVANCEMENT OF MEDICAL SCIENCE.

(Delivered before the Esculapian Society, at its Semi-Annual Meeting, in Marshall, Ill., May 26th, 1858.)

BY O. Q. HERRICK, M. D.

*Mr. President, Members of the Esculapian Society, Ladies and Gentlemen:*

At no period in the history of the human race has there been such efforts made to ameliorate its condition as at the present time. The pages of ancient history contain little but records of oppression, persecution and degradation of the masses, to satisfy the love of power and lust of ambition of pampered monarchs, who deemed no measures too humiliating in their character, or demoralizing in their tendency, to be used in the accomplishment of their selfish purposes. And so it must ever have remained, had not some mighty mind, conscious of its inherent greatness, arose and broke the shackles of ignorance and superstition, and boldly declared the image of God too noble a thing to be made subservient to the idle dreams and caprices of despotic power.

To mankind this truth came like sunbeams bursting from the cave of night. All felt its healing influence, and not a few hailed it as the morning star of intellectual freedom, and nurtured it with the power of their being. Virtuous principles and lofty aspirations could no longer be kept in bonds, or quieted in dumb servility. There was a mysterious longing in thinking minds to explore the endless fields of thought, and gather food upon which they might be nourished and brought to maturity.

Thoughts of God, the great first cause, and the beauty of the world, of necessity first occupied the attention of original thinkers, and conceptions formed early, found an expression which introduced the creative art. Crude and imperfect as it first appeared, kings threw their protection around its votaries, and opened the public coffers for their benefit. Ideal forms of beauty not attained spurred on to renewed exertion, until lips were made to quiver in marble, eyes to laugh upon canvas, and "forms of matchless symmetry brought forth, which must charm and instruct forever."

But while hosts of admirers were vieing with each other in lauding the various works of art, there were a few of towering genius and wondrous thought, who labored incessantly to acquaint themselves with the "cunningest part of excelling nature." With no truths by others gained, or facts established, it required unswerving stability, untiring perseverance, and an imperishable desire to relieve human ills, to enable these explorers in a newly-opened philanthropic field, to relinquish the claim to fame, honor and wealth, which their natural endowments would have demanded for them in the artist's ranks, and unsolicited and unaided, spend a life of toil, endeavoring to establish means by which the enemies of health may be successfully met and driven back from the threshold, that otherwise must supplant the pleasures of home with sorrow and tears.

As we might expect, from the circumstances by which they were surrounded, and the multiplicity of disadvantages under which they labored, facts accumulated but slowly, and for a long period bore but a small proportion to the number of erroneous views entertained with regard to the origin and treatment of disease. But, few as they were, enough were elicited to satisfy earnest inquirers after truth, that close research and observation, with careful experiments, would establish a science calculated to soften the pangs of disease and prolong life.

The people, accustomed to see the pestilence nearly depopulate portions of the country and leave it an abandoned waste, that had witnessed the plague's unmolested march as it swept away the flower of their youth, and left desolation and distress in its track, were ready to shower honors upon any one who should devise means by which to turn away the blighting hand that so often stole into the family circle and sapped the sweets of social life.

As soon as the public eye was turned upon the subject, a crafty priesthood, ever eager for notoriety where fame cannot be reached, whose only knowledge of anatomy and physiology consisted in a belief that man was fearfully and wonderfully made, commenced anew their work, and incessantly toiled to convince a credulous and grossly ignorant populace that all diseases were only manifestations of God's wrath, and offered for their cure remedies which could only be found in the wildest dreams of fancy.

While others, afraid of being left in the rear, hastily bundled together a few facts, combined with them presumptuous assertions, and set the world agog with ideas of atoms and pores, elements and ethers, and mystified and enshrouded the little known, until medicine appeared inextricably mixed up with priestcraft and intolerable superstition.

The time at length arrived when this night of ignorance could last no longer. There came upon the stage a man of intellect almost infinite. With "perception vast and reason deep," he observed the working of nature in her different abodes, and saw contending forces destroy her brightest ornaments, and crumble her choicest gems. United and concentrated efforts reversed the work, and brought out her valued and beautiful productions.

He determined to apply the prompting thus received to the establishment of a science which should embrace the laws of health and disease. To consumate this purpose, it became necessary to collect the varied opinions of his antecedents and cotemporaries in the art, when, by close research and comparison, he formed his conclusions, expunged the fallacious notion of supernatural causes operating upon the body, overthrowed the doctrines of priestcraft, and established upon the immutable pillars of truth the science which has often dried the orphan's tears, stilled the widow's cry, and poured the oil of gladness around the hearthstone of discontent.

It was no great original discovery of this physician of Cos in medical science that wrought such a marvelous result; but gathering in one body the opinions of others, and applying to them the inductive mode of reasoning, was the great work which sealed his name for immortality.

A history of this noblest science, however brief, cannot be given in the short time alloted us. We first view it as a narrow stream, almost inaccessible on the heights of antiquity, and as it rolls forward, is at times almost lost among cascades and shadowy coverts; but in time it reaches the plains of historic truth, and flows onward, a majestic river, securely embanked by written documents and recorded facts.

I am aware that the idea is frequently entertained, especially among the junior members of the profession, that all that is valuable in medicine has originated in the nineteenth century, which is far from being true. In the writings of Galen and Hippocrates, though their ideas were crude and imperfect, we find guiding principles that must ever remain pillars of wisdom, which the waves of charlatanry cannot weaken, the syrian winds of quackery cannot break down, and whose strength trembles only at the tread of omnipotence.

A constant desire for change is characteristic of the present day, but changes have not in every instance benefited mankind or advanced the profession. That the science of medicine is now advancing more rapidly than at any former period I have no doubt. This is accounted for by the more general recognition of the old axiom, that in union there is strength.

The genius of our science is such, that with the increased aid of collateral sciences it is impossible for us to remain satisfied with our present attainments. Our aim is too high, the objects to be accomplished too noble, I may say sacred, in their character to permit us to ever flag or unproperly apply our time until disease is shorn of its terrors, and those maladies which have long resisted our attempts to cure, shall come crouching to our feet. This work can never be fully completed without unremitting toil and unity of action. Mind, no less than matter, gains new form and force by consociation. It is but recently that the masses have heeded the lessons of unintelligent creation. The glistening dewdrops that so lightly fall and quickly disappear, when gathered, make the soundless ocean that moves with majestic mien before the tempest car, and glories in its strength.

So with thought. Each active mind may generate sound practical ideas, that, to remain only within the author's province, would be like a well-shaped stone for the builder's use, useless alone; but when put in its proper place, when linked with the reasoning of other minds, may change the course of empires, or, perhaps, open up a science that otherwise might have slumbered for ages.

The desire of rousing the energies of skilful men, and increasing the number of offerings for the public good, of opening to its full width the channel which leads to the great foundation of benevolence, alone led to the formation of this and kindred associations, and the benefits that accrue, not only to the members composing them, but to the recipients of their care, are incalculable. In these are found the ablest and most valued talent in the profession. The almost constant desire of the young for innovation is here checked by the aged, and ideas of having mastered the science vanish while listening to the counsel of those who have spent long lives in scanning the effects of remedies in their different combinations, under the various circumstances that modify their action, and who have witnessed the guarded and insiduous, no less than the demanding attack of disease as it searched for the secret spring of life. The aged are here stimulated by the ardor of the young, and are cheered on by seeing them bring rich offerings from fields which the sage teachings of time had not disclosed. Cases of peculiar interest, with the success attending a certain course of treatment, that never would appear in the journals of the day, are here given in detail, and whatever of value is in them is preserved for the public good. That professional jealousy which frequently proves an impassable barrier between worthy men is here subdued, and the withered hand of friendship regains its form and usefulness. Wavering confidence in a brother's fidelity is here lost, by witnessing his devotion to correct principles, and instead of passing him with a suspicious, silent nod, we pay respect to his claims, and meet him as "friend meeteth friend."

No class of men so much as ours are tempted to question the ability and doubt the honesty of their co-workers. We are almost daily told of somebody that is made a cripple for life by injudicious treatment, of some patient that was killed by too much medicine, or, of some other that died for want of more, which, with the wonderful things our informants "know of," are calculated to lead us to believe that some doctors are not any better than they should be. And was not this officious impudence of talebearing confined exclusively to the unrespected members of society, it would do more harm. It is due not only to ourselves, but to our patients, to whom they are ever ready to offer their sickly tattle and advice, that these meddlers in such matters, these mildewed specimens of humanity, should receive such rebuke as their meanness demands.

It is only by association that we gain a correct estimate of each other's merits, and he who refuses measurement by this standard, can hardly presume to stand above suspicion. If at times we differ upon certain points, there is no place where truth is so likely to be elicited as here, and however warm and animated the discussions may be, new ideas are sure to spring out of them, and instead of arousing a spirit of animosity between the contestants, there springs up a feeling of warmer friendship and increased respect. It is here that the dormant energies are called into action, and sleeping indolence is entombed by the active engagement of the nobler powers of the mind.

By liberal contributions from members situated in different localities, that have subjected disease to different modes of treatment, and carefully noted the result, medical societies advance rapidly the cause for which they were instituted, and will eventually become the great reservoir of thought, in which must spring up the "balm for the healing of the nations."

Associations for the purpose of advancing medical science are now becoming deservedly popular, and receive as they merit the consideration of those who are desirous of improving themselves, or advancing the best interests of the community in which they live. There are those engaged in medicine, however, that can receive no benefit from association. And we will here say to you who have the whole science embodied in your breast, but have no means by which we can be made cognizant of the same, with no desire of increasing your stock of knowledge, better not spend your time in meetings like this. We can be of no service to you, and I fear that we, like your patients, will seldom be benefited by your presence. But to you who are not satisfied by treading only in paths worn down by your fathers, who are desirous of quieting the maelstrom of unhappiness and completing the temple to which all may look and live, we say, "Come, go with us, and we will do you good." We ask you to unite with us, not merely to increase our members, but as co-laborers. Spending none of your time in idle retreats, but subjecting yourselves to constant attrition of mind, that our usefulness may be increased, and we assure you your efforts shall not go unrewarded. We ask you to study well the judicious use of long known remedies rather than the list of doubtful, untested substitutes.

Every "ignis fatuus" that appears in the medical horizon should not be hunted down by the regular practitioner. To be induced to leave established landmarks in the profession by the vaunting promises that cluster around new theories and speculative practice, would be selling a birthright in standard medicine for an equivalent of less value than Esau's pottage.

System after system of quackery has originated in the idle dreams of some brainless mountebank, and each, like Jonah's gourd, for a time has grown, and, like it, died. Their scions are always transplanted in shallow ground, which, for a time, yields them a support, but they ultimately droop, die, disappear, and are forgotten.

We gain an instructive lesson from the effects which the simoons of empiricism have produced in the last few years. Willing martyrs have appeared to test the efficacy of every remedy clothed with the charm of mystery. Some have been swathed in cold sheets, endeavoring to freeze out the intermeddlers of health, while others have made fruitless attempts to accomplish the same by being boiled. In common with other subjects of quackery, after a few trials, they have generally died, which, to themselves, their families and friends, was the kindest act they could perform. We clearly see that ignorance is the great hot-bed in which these pests find an origin, and is their only source of nourishment. When this shall be dispelled, when the masses manifest the same interest in medicine as politics, the various *isms* and disordered growths, more to be dreaded than Banquo's ghost, that are vainly searching for a resting place, shall collapse and disappear like the "baseless fabric of a vision."

As conservators of the public weal, we are bound by every principle of humanity not only to warn the public against imposition from outsiders, but to guard against the possibility of their finding unsafe practitioners in the regular profession. This is a matter that we have too long entrusted to the public schools. It is time that we should take a stand point and demand a searching investigation into the qualifications of every man with whom we hold fellowship.

Our generous deportment and universal adherence to truth, have led the masses to believe that none but honest men would wear the garb of our profession. Hence it is that the indigenous, nondescript productions, called eclectics and Indian doctors, have found employment until their history is written in our graveyards, and in vacant seats in the family circle. We should not be anxious to introduce into practice any man because he appends to his name M. D. in flaming characters.

Acquaintance may convince us that it is necessary to consider them the initials of miserable deception, to describe his character as a physician.

Many of our schools vie with each other in the rapidity of making doctors, and have long struggled, "Jacob like," to see who could confer degrees upon the aspirant with the least fitness to discharge the various duties incumbent upon the practitioner of medicine. No one thing has done more to foster empiricism than this. But one school in the West has made an effort to come up to the recommendations of the American Medical Association, and that deprived of clinical advantages, however brilliant and numerous may be its corps of professors, must ever fail of accomplishing its mission. Private practitioners as a body must demand a higher grade of medical education. Then, and not till then, will our schools increase their usefulness.

It is useless for us to look along the Atlantic coast to guide us through the intricate windings of our profession; there peers up in the Northwest some modest stars that are much better guides through the fogs and mists of the Wabash Valley than any which have yet appeared. We have men no less noted for their superior attainments in medicine than our western country for its natural advantages; men, upon whom nature has bestowed her choicest gifts, to whom science looks for additional strength. By their fine discrimination and accurate reasoning, our State is furnished with a school above the common grade, and fully up to our demands, but not our wants. The fault is our own that its character is not more elevated.

We are leagued with the public teachers in preparing men with whom we are bound to strike hands as brothers to labor in one common cause, and should not expect them to make skilful men unless we furnish them with well trained, industrious students.

We should be careful that none enter our offices to pursue the study of medicine without previous mental discipline, without literary and scientific acquirements sufficient for a commanding position in society, and ask our schools to confer degrees only on men of intelligence, well skilled in the various branches that aid in tracing disease to its origin, or assist in its removal. Unity of sentiment and action is here only necessary to the speedy accomplishment of a radical change in the profession, and nothing is more desirable, or could be productive of better results.

But elevate the standard of medicine, and it becomes robed with additional lustre, its supporters gain additional confidence from the subjects of their care, and the dew of its blessings shall be cheering to the fainting hopes of health, as gentle rain to drooping flowers. No former period has promised so bright a future as the present. Association, as an element of success, is not only being felt and acknowledged, but the principle is being put into practice. Its teachings are blotting out the errors, and dispelling the shadows which have surrounded us, and are building on life's most troubled coast a lighthouse that shall prove an unerring guide to the welcome shores of health.

As our aim is higher than that of other callings in life, let us be more devoted, industrious and persevering. The zeal of the true physician glows in the crucible of adversity, no less than when rocked in the golden chair of plenty. The sacrifice which we are required to make to fully do our duty, can never be prompted by the grovelling whispers of avarice. 'Tis not for wealth that we would leave the pleasures of a quiet home and spend our time where tales of sorrow only are told. 'Tis not for this that we would spend sleepless nights where the cry of suffering wounds deep the finer feelings of the heart. 'Tis not for this that midnight finds us visiting the unfortunate poor, where deadly vapors load the air, and death stalks forth unmasked. 'Tis not because we know the name of the good physician is engraven upon the tablets of memory, and shall live on when the names of martial heroes shall be buried deep in forgetfulness. But it is a knowledge of performing the noblest work on earth, by bearing away many of the sorrows of life, and replacing them with the benison of gladness, and the hope of finally bringing under subjection all diseases which mar the happiness of the human family. This is the buoying hope of of our profession; and no class more than ours will rejoice to see the day when the sounds of suffering shall fall upon the air no more, when the blush of health shall mantle every cheek, and the whole pathology of life, from the days of early innocence to ripe old age, shall be thickly strewed with the rose leaves of pleasure and peace.

